# Retinoic Acid Profiles in Proliferative Verrucous Versus Homogeneous Leukoplakia: A Preliminary Nested Case–Control Study

**DOI:** 10.3390/biomedicines13081881

**Published:** 2025-08-02

**Authors:** Cintia M. Chamorro-Petronacci, Alba Pérez-Jardón, Susana B. Bravo, Pilar Gándara-Vila, Andrés Blanco-Carrión, Yajaira Vanessa Avila-Granizo, Alejandro I. Lorenzo-Pouso, Sara A. Prieto-Barros, Mario Pérez-Sayáns

**Affiliations:** 1 Oral Medicine, Oral Surgery and Implantology Unit (MedOralRes), Faculty of Medicine and Dentistry, Universidade de Santiago de Compostela, Entrerríos s/n, 15705 Santiago de Compostela, Spain; cintia.chamorro@usc.es (C.M.C.-P.); pilar.gandara@usc.es (P.G.-V.); andres.blanco@usc.es (A.B.-C.); saraprietobarros@hotmail.com (S.A.P.-B.); mario.perez@usc.es (M.P.-S.); 2 ORALRES Group, Health Research Institute of Santiago de Compostela (IDIS), 15706 Santiago de Compostela, Spain; 3 Proteomic Unit, Health Research Institute of Santiago de Compostela (IDIS), University Clinical Hospital of Santiago de Compostela, Travesía da Choupana S/N, 15706 Santiago de Compostela, Spain; sbbravo@gmail.com; 4 College Dentistry, University of Guayaquil, Guayaquil 091910, Ecuador; yajaira.avilag@ug.edu.ec; 5 Instituto de los Materiales de Santiago de Compostela (iMATUS), Avenida do Mestre Mateo, 25, 15782 Santiago de Compostela, Spain

**Keywords:** retinoic acid, mouth neoplasm, oral leukoplakia, proliferative verrucous leukoplakia, mass spectrometry

## Abstract

**Background**: Oral leukoplakia (OL) and proliferative verrucous leukoplakia (PVL) remain challenging entities due to the absence of reliable prognostic biomarkers. All-trans retinoic acid (atRA), a pivotal modulator of epithelial differentiation and mucosal integrity, has been proposed as a candidate biomarker. This study sought to quantify plasma RA levels in patients with OL and PVL compared to healthy controls, assessing their potential clinical utility. **Methods**: A cohort of 40 participants was recruited, comprising 10 patients with OL, 10 with PVL, and 20 healthy controls. This nested case–control study was derived from previously characterized institutional databases of oral potentially malignant disorders. Plasma samples were analyzed for atRA concentration using high-precision mass spectrometry. Statistical comparisons were conducted to evaluate differences between groups and associations with clinical outcomes. **Results**: Patients with homogeneous OL exhibited significantly reduced plasma atRA concentrations (mean 2.17 ± 0.39 pg/mL) relative to both PVL patients (2.64 ± 0.56 pg/mL) and healthy controls (2.66 ± 0.92 pg/mL), with *p*-values of 0.009 and 0.039, respectively. No statistically significant difference was found between PVL patients and controls. Furthermore, atRA levels demonstrated no correlation with clinicopathological variables or malignant progression within the PVL cohort. **Conclusions**: These preliminary findings indicate that diminished plasma atRA levels may serve as a prognostic marker for homogeneous oral leukoplakia, whilst its role in PVL appears limited. However, effect estimates were imprecise, and additional studies are warranted.

## 1. Introduction

Oral leukoplakia (OL) is the most common oral potentially malignant disorder (OPMD), with global prevalence estimates ranging from 1.36% to 4.11% [1]. At the International Seminar on Nomenclature and Classification of OPMDs held in Glasgow in 2020, convened by the World Health Organization (WHO) Collaborating Centre for Oral Cancer, OL was defined as “a predominantly white plaque, after exclusion of other known diseases or disorders that do not carry an increased risk of cancer” [2]. Histopathologically, OL is characterized by epithelial hyperkeratosis, hyperplasia, and dysplasia of varying degrees.

From an epidemiological standpoint, OL affects an estimated two to three million individuals worldwide each year. Prevalence varies significantly across regions, largely reflecting differences in exposure to established risk factors [3]. Tobacco use remains the most well-established causal factor, with recent systematic reviews providing robust evidence of a clear dose–response relationship between smoking intensity and OL risk [4]. Additional contributing factors include alcohol consumption, particularly when combined with tobacco, and betel quid chewing, which is especially prevalent in South and Southeast Asia [5].

Data from the WHO Collaborating Centre indicates that OL has a malignant transformation rate ranging from approximately 7.9% to 11.7% [6], with higher rates observed in Western populations and patients referred to tertiary care centers. Although OL is often asymptomatic [7], its potential progression to oral squamous cell carcinoma (OSCC) poses a significant clinical concern. Several factors have been identified that increase this risk, including non-homogeneous lesion morphology, high-grade epithelial dysplasia, and lesion size exceeding 200 mm2 [8]. Patients with OL experience a significant psychological burden involving cancer-related anxiety and reduced quality of life [9].

Proliferative verrucous leukoplakia (PVL), previously regarded as an aggressive variant of OL, is now recognized as a distinct clinicopathological entity [10]. According to WHO criteria, PVL is defined as “a distinctive form of oral leukoplakia characterized by multifocal white lesions that tend to become exophytic and verrucous over time, with high rates of recurrence and malignant transformation” [2]. PVL is characterized by high recurrence rates following treatment, estimated at 67.2%. PVL exhibits an exceptionally high malignant transformation rate, reaching up to 70% [11]. Epidemiologically, PVL predominantly affects elderly women, with a mean age at diagnosis of 70.2 years [12]. Unlike conventional OL, PVL appears largely independent of tobacco and alcohol exposure [13].

Early-stage PVL is notoriously difficult to diagnose, as its lesions are clinically and histologically indistinguishable from ordinary leukoplakia [14]. PVL typically progresses from flat white patches to multifocal, exophytic, wart-like lesions that are resistant to standard treatments [15]. This unique clinical course, coupled with its aggressive behavior, necessitates tailored management strategies, including more frequent monitoring and a more assertive therapeutic approach [16].

Both OL and PVL are believed to have multifactorial and largely unresolved etiologies. Increasing attention has focused on their underlying genetic and epigenetic drivers, particularly chromosomal instability [17]. Notably, mutations in the TP53 and KMT2C genes have been implicated in the malignant progression of OL [18]. However, no molecular marker has yet demonstrated sufficient reliability for clinical use as a diagnostic or prognostic biomarker for either OL or PVL [19].

All-trans retinoic acid (atRA), especially its 13-cis isomer (isotretinoin), has shown promise in treating oral premalignant lesions and preventing second primary tumors in patients with OSCC [20]. The metabolite of vitamin A, atRA, plays a critical role in regulating cellular differentiation, immune responses, and epithelial turnover [21]. Over several decades, clinical trials have reported complete response rates ranging from 10% to 27% and partial response rates between 54% and 90% following topical or systemic retinoid treatment [22]. AtRA mediates its effects through a family of nuclear receptors—retinoic acid receptors (RARs α, β, and γ) and retinoid X receptors (RXRs α, β, and γ)—which function as ligand-dependent transcription factors [23,24]. Among these, RAR-β2 is of particular interest; this putative tumor suppressor is frequently silenced in oral premalignant lesions and head and neck cancers, often via epigenetic mechanisms such as promoter hypermethylation [25]. RAR-β expression has also been inversely correlated with cyclooxygenase-2 (COX-2) levels, suggesting that atRA may modulate tumor risk through anti-inflammatory pathways [26].

Vitamin A deficiency has long been associated with human oncogenesis [27], and genetic polymorphisms affecting atRA metabolism have been linked to increased cancer susceptibility [28]. Specifically, variants in CYP26A1 and CYP26B1—key enzymes involved in atRA catabolism—have been shown to increase the risk of oropharyngeal cancer, independently or synergistically [29]. Elevated activity of these enzymes can accelerate atRA degradation, reduce its bioavailability, and potentially diminish its protective effects. Indeed, higher levels of atRA-degrading metabolites have been detected in cancer patients, which may impair cellular apoptotic responses and facilitate malignant progression [30]. In a previous study, our research group investigated a three-generation family comprising 17 individuals, of whom three developed OSCC and one developed esophageal adenocarcinoma, all in the absence of traditional risk factors such as tobacco and alcohol. Genomic analysis identified a CYP26B1 polymorphism (2p13.2; G>T) in affected individuals. Subsequent serum analysis revealed a consistent association between this variant and reduced circulating atRA levels [31].

Building on this evidence and our prior findings, the primary objective of the present study is to investigate the relationship between serum levels of all-trans retinoic acid (atRA) and the diagnosis and prognosis of two oral potentially malignant disorders: homogeneous oral leukoplakia and proliferative verrucous leukoplakia. We hypothesize that atRA dysregulation may contribute to the pathogenesis of these conditions and serve as a potential biomarker for risk stratification.

## 2. Materials and Methods

### 2.1. Subjects of Study

This study was designed as a nested case–control pilot study. Ethical approval was obtained from the Santiago-Lugo Research Ethics Committee (Ref: 2018/503). This study adhered to STROBE guidelines for reporting observational studies [32]. Written informed consent was obtained from all participants in accordance with ethical guidelines and the Declaration of Helsinki and subsequent amendments. We conducted a nested case–control study using participants identified from institutional databases of patients with homogeneous oral leukoplakia and proliferative verrucous leukoplakia (PVL), which had been previously audited, characterized, and published by our research group in earlier investigations [14,33].

The case groups included 10 patients clinically diagnosed with oral leukoplakia (OL) and 10 patients with proliferative verrucous leukoplakia (PVL), all recruited at the Oral Medicine Unit of the Faculty of Dentistry, University of Santiago de Compostela (Coruña, Spain) (Table 1). The diagnosis of OL and PVL was established in accordance with the World Health Organization (WHO) criteria for oral potentially malignant disorders (OPMDs) [2]. Specifically, OL was defined as a predominantly white, non-removable lesion of the oral mucosa that could not be characterized clinically or pathologically as any other definable condition [33]. PVL, in contrast, was diagnosed based on its multifocal presentation, persistent and progressive nature, and characteristic clinical evolution over time, including resistance to treatment and tendency toward malignant transformation, as described in WHO guidelines and major clinical classification systems [14].

**Figure 1:**
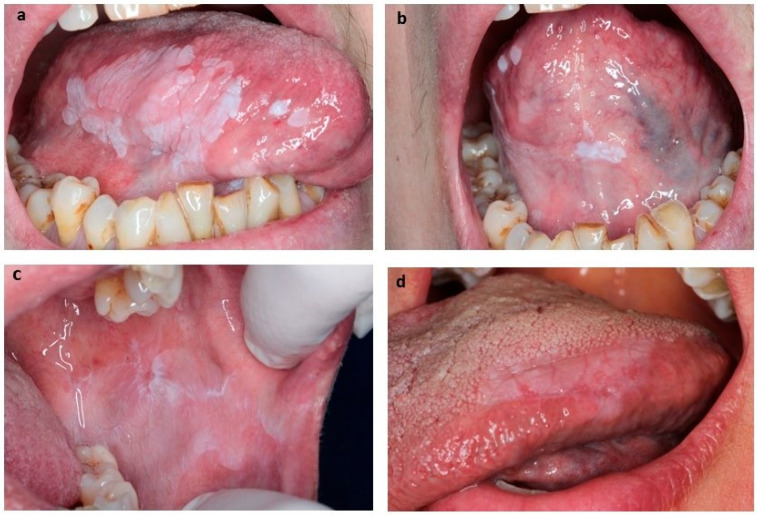
Clinical images of Oral Leukoplakia (OL) and Proliferative Verrucous Leukoplakia (PVL). (**a**) Extensive PVL on the right tongue side. (**b**) PVL in the region of the intersection of the lingual frenulum on the ventral surface of the tongue. (**c**) Homogeneous OL in left buccal mucosa. (**d**) Homogeneous OL on the right-side edge of the tongue.

To support and validate the clinical diagnoses, all cases underwent clinicopathological correlation, with confirmation through histopathological evaluation. The general diagnostic categories are summarized in Table 2. Additionally, clinical presentations are described in detail, including their anatomical location and corresponding pathological findings in Table 3. Histological findings ranged from epithelial hyperkeratosis to varying degrees of oral epithelial dysplasia (OED), with three PVL cases presenting severe dysplasia that subsequently progressed to malignancy. The three-tier system of oral epithelial dysplasia (mild, moderate, severe) was used [33]. Only patients who had not received surgical excision, laser vaporization, or any other specific treatment since diagnosis were included in the follow-up cohort to assess the natural clinical progression of the lesions. All patients underwent standardized, long-term follow-up through regular clinical examinations, with a mean follow-up period of at least 10 years. Representative clinical images of OL and PVL lesions are shown in Figure 1.

A total of 20 healthy individuals, matched by sex and balanced by age (±5 years), were included as the control group. These participants were recruited from the Integrated Adult Dentistry Service of the USC Faculty of Dentistry and Vithas Vigo Hospital (Vigo, Pontevedra, Spain) during routine dental or medical appointments for conservative dental treatments or general health check-ups. All patients received, and continue to receive, standard care based on their individual clinical needs, including routine monitoring of lesions irrespective of whether they had undergone surgical excision or laser vaporization [14]. For the purposes of this study, only those cases in which no additional treatment for OL or PVL had been administered since the time of diagnosis were classified as being under “follow-up”.

### 2.2. Sample Size Calculation

Total sample size was estimated a priori by considering the results from a previous family study analyzing atRA levels in OSCC patients, reporting mean concentrations of 1.67 ± 0.49 pg/mL in patients who developed malignization versus 4.25 ± 1.56 pg/mL in those who did not (*p* = 0.03) [31]. G*Power 3.1.9.7 was employed for calculation; specifically, these values were input in the family *t*-tests for means of two independent groups (two-tailed). Using an effect size of d = 2.08 (calculated from the means and pooled standard deviation), a total of 6 participants per group would have provided a power of 0.8 with an α value of 0.05. However, considering the pilot nature of this study and potential sample losses, the sample size was increased to 10 participants per group for the OL and PVL groups, with 20 healthy controls to ensure adequate statistical power and to prevent plausible data attrition.

### 2.3. Post-Hoc Power Analysis

Given the observed effect sizes in our study, post hoc power analysis revealed that our sample achieved >80% power to detect the observed differences between OL and control groups (Cohen’s d = 0.67) and between OL and PVL groups (Cohen’s d = 0.98). While these effect sizes are smaller than initially anticipated based on the preliminary data from our previous study [31], they represent clinically meaningful differences in the context of biomarker research for OPMDs.

### 2.4. Collection and Treatment of Blood Samples

Two tubes of peripheral venous blood were obtained from each patient. To prepare the samples, the tubes were centrifuged at 4000 rpm for 15 min (Analogue Angular Centrifuge, Quirumed brand (Valencia, Spain; CNT800D). Next, only the plasma was pipetted and immediately frozen at −20 °C for conservation. This methodology was previously described by our group [34].

### 2.5. atRA Extraction Process

This phase of the procedure was carried out in the Proteomics service of the Health Research Institute of Santiago de Compostela (Coruña, Spain). The procedure outlined in the article by Kane et al. (2005) was followed [35]. All samples were processed in duplicate to ensure reliability, and the laboratory personnel were blinded to group assignment to minimize bias. Briefly, 200 μL of the serum from each sample was added to a disposable glass culture tube (16 mm × 150 mm), together with a volume of 15 μL of the internal pattern (50 nM 4,4-dimethyl RA in acetonitrile). Moreover, 1 mL of MKOH in ethanol was incorporated and mixed by vortex. Next, 10 mL of hexane was added, vortexed again, and the samples were centrifuged at 60 rpm for 1–3 min to achieve phase separation. Once these phases were obtained, the hexane layer, which contained lipids (including retinol and retinyl esters), was removed. A 60 μL amount of 4 M HCl was added to the aqueous phase and mixed with vortex, followed by the addition of a volume of 10 mL of hexane and again mixed with vortex, after which it was centrifuged under the same guidelines described above. The phases were separated, and the solvent from the second hexane phase (containing atRA) was removed under a gentle stream of nitrogen at 25–30 °C, with special care to avoid contamination of this hexane layer with the acidic aqueous layer (which would catalyze the isomerization of atRA). The hexane phase was dried under vacuum in a speedvac (miVAC, Fisher Scientific, Loughborough, UK) at 36 °C. The resulting residue was dissolved in 60–100 μL of acetonitrile and taken to a deactivated glass autoinjector for subsequent analysis [35].

### 2.6. atRA Quantification

To carry out this phase, the participation of the Research and Technological Development Support Infrastructure Network Mass Spectrometry and Proteomics Service was required (Coruna, Spain). The same study methodology explained in the article by Pérez-Sayáns et al. was followed [31]. For mass analysis, mass spectrometry technology with a triple quadrupole analyzer was used. API 4000 equipment (AB SCIEX, Framingham, MA, USA) was used, coupled to an HPLC from Agilent (Santa Clara, CA, USA) model 1200RR. The ionization source was APCI POSITIVE, the source temperature was 350 °C, and the oven temperature was 30°. The sample was resuspended in 50 μL of ACN.

A 20 μL amount of each atRA sample obtained in the process explained in the atRA extraction process section was used. Using this method, levels of atRA (namely, all-trans retinoic acid) were measured.

### 2.7. Statistical Analysis

A dedicated database was established to record the following variables: sample group (homogeneous OL, PVL, control), sex (male/female), age, tobacco consumption (cigarettes per day), alcohol consumption, date of OL/PVL diagnosis, malignancy status (yes/no) and date of diagnosis if applicable, malignancy location, date of last clinical follow-up, treatment modality (follow-up, laser, excision), histopathological findings and atRA concentration (pg/mL) for each participant.

Statistical analyses were conducted using SPSS version 28 (IBM Corp., Armonk, NY, USA). Data were summarized as frequencies and percentages for categorical variables and means with standard deviations for continuous variables. Normality was assessed using the Shapiro–Wilk test. Associations between categorical variables were evaluated using Chi-square tests with appropriate corrections. Differences in means among groups were analyzed using one-way ANOVA followed by Bonferroni post hoc correction for multiple comparisons. Pearson’s correlation coefficient was employed to assess relationships between continuous variables. Receiver Operating Characteristic (ROC) curve analysis was performed to assess the discriminative ability of calculated concentration values to differentiate between the three groups. The Area Under the Curve (AUC), sensitivity, specificity, positive predictive value (PPV), and negative predictive value (NPV) were calculated at optimal threshold points. Statistical significance was set at *p* < 0.05.

## 3. Results

### 3.1. Clinical and Follow-Up Findings

Of the 40 initial participants, four samples (1 OL, 3 controls) were excluded due to being below the quantification range. Therefore, 36 samples were analyzed: 19 with OL or PVL and 17 healthy controls. The descriptive data of the patients can be seen summarized in Table 1.

The mean age of patients at the time of blood sample collection was 64.28 ± 12.27 years (range: 44–87 years). The clinical and histopathological diagnoses of OL and PVL were established at a mean age of 58.79 ± 12.12 years (range: 39–80 years). Among the 36 samples analyzed, malignant transformation was observed in three patients, all with a history of PVL. These cases comprised two males and one female, with malignant transformation occurring at a mean age of 62.67 ± 12.70 years (range: 48–70 years). The malignant lesions arose at distinct oral sites, including the gingiva, buccal mucosa, and alveolar ridge. Only one of these patients was a smoker (20 cigarettes/day), who had ceased tobacco use after diagnosis. All patients were T1N0M0 at the OSCC diagnosis time point. Additional histopathological data are provided in Table 2.

### 3.2. All-Trans Retinoic Acid Levels

The mean atRA concentrations were 2.17 ± 0.39 pg/mL in the homogeneous OL group, 2.64 ± 0.56 pg/mL in the PVL group, and 2.66 ± 0.92 pg/mL in the control group. Statistical analysis revealed no significant associations between atRA levels and sex (*p* = 0.918), tobacco consumption (*p* = 0.838), alcohol consumption (*p* = 0.421), or malignancy status (*p* = 0.276). A detailed summary of the associations between atRA concentrations and clinical variables is presented in Table 3.

One-way ANOVA analysis revealed that the mean concentration of OL samples was significantly lower than that of control group samples (*p* = 0.039). This difference was maintained when comparing OL to PVL, meaning that the atRA concentration in patients with OL was also significantly lower than in patients with PVL (*p* = 0.009).

**Figure 2:**
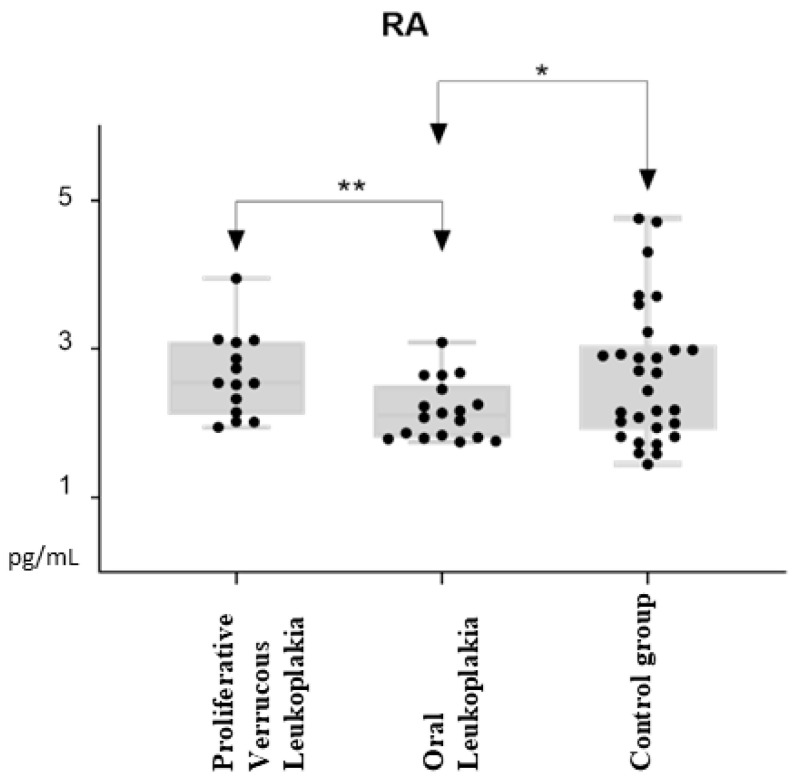
Dispersion of atRA concentration values (pg/mL) in box and whisker plot. * *p* < 0.05, ** *p* < 0.01.

However, no significant differences were observed between the PVL and the control group samples (*p* = 0.953). In Figure 2, the dispersion of the results is shown by groups.

**Figure 3:**
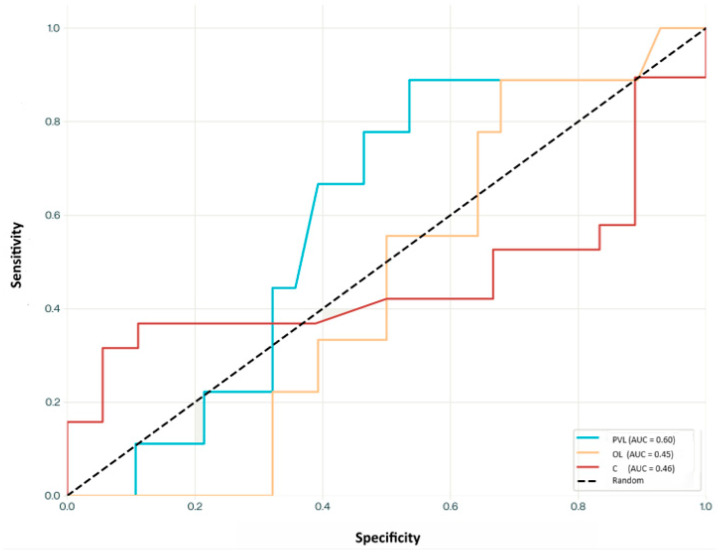
ROC curves for proliferative verrucous leukoplakia, leukoplakia, and control groups calculated using atRA concentration.

ROC curve analysis was performed to evaluate the diagnostic potential of the calculated concentration for distinguishing between the three groups (Figure 1). The PVL group showed the highest discriminative capacity with an AUC of 0.60, although this represents only modest diagnostic ability. Both OL and control groups demonstrated poor discriminative performance with AUC values of 0.45 and 0.46, respectively, indicating worse-than-chance performance. At the optimal threshold for the PVL group (2.33 ng/mL), sensitivity was 0.67, and specificity was 0.60. For the OL group, the best threshold (2.14 ng/mL) yielded a sensitivity of 0.56 and specificity of 0.40. The control group showed its optimal threshold at 2.08 ng/mL, with a sensitivity of 0.43 and a specificity of 0.51 (Figure 3).

## 4. Discussion

A total of 36 samples from patients with OL, PVL, and a control group were included in this study. It was observed that the concentration of atRA in patients with OL was significantly lower than in the control group, and this difference was maintained when compared to the group of patients with PVL. However, no significant differences were observed between the group of patients with PVL and the control group. These results suggest a relationship between atRA concentration and the type of oral lesion, which could have important implications in the clinical management of these disorders.

This pilot study provides preliminary evidence that patients diagnosed with homogeneous oral leukoplakia exhibit significantly reduced serum concentrations of all-trans retinoic acid (atRA), with an observed 18% decrease relative to healthy controls and a 22% decrease when compared to patients with proliferative verrucous leukoplakia.

While the absolute differences in atRA concentrations between groups may appear modest (0.47–0.49 pg/mL), these represent proportionally meaningful alterations when considered within the context of vitamin A metabolism. The 18–22% reduction in atRA levels observed in OL patients compared to controls and PVL patients represents a biologically relevant shift that could significantly impact epithelial homeostasis over time. Retinoic acid operates within narrow physiological ranges, and even small perturbations can have cascading effects on cellular differentiation, proliferation, and DNA repair mechanisms [36].

The consistency of our findings with previous research [31], which reported significantly lower atRA levels in OSCC patients (1.67 ± 0.49 pg/mL), suggests the existence of a progressive decline in RA signaling throughout the stages of oral carcinogenesis: healthy controls > PVL ≈ controls > OL > OSCC. Although no significant differences were observed between PVL and control samples in our study, this pattern raises the hypothesis that retinoic acid (RA) depletion may represent an early molecular event in oral carcinogenesis, potentially initiating before overt morphological changes become detectable by conventional histopathology [37]. It is possible that, in PVL, RA levels remain unaltered until a later stage of malignant transformation, or that, alternatively, possibly genetic pathways predominate in its pathogenesis. The lack of significant differences in atRA levels between PVL samples and healthy controls in our study may reflect the distinct etiopathogenesis of PVL, which appears to be less strongly associated with modifiable risk factors.

In this context, the concept of field cancerization may also be relevant. In conventional OL, chronic exposure to carcinogens such as tobacco and alcohol is thought to induce widespread molecular alterations across the oral mucosa, including depletion of RA signaling. By contrast, PVL may represent a separate pathogenic process, less influenced by environmental exposures and more driven by intrinsic, possibly genetic, factors. This could explain why PVL does not exhibit the same RA depletion profile as OL, despite its high malignant potential.

Regarding the clinical variables studied, the atRA concentration does not seem to present significant variation depending on the sex of the patient. On the other hand, when the data were analyzed by study groups associated with sex, men with OL have a lower RA concentration than women, although it is not statistically significant (*p* = 0.091). This aspect would be consistent with the fact that the incidence of OSCC in men is double the incidence in women, although this difference has been decreasing over the years 20. Furthermore, these results coincide with the findings of Pérez-Sayáns et al., in which they also found no significant differences between sex with respect to atRA concentration (*p* = 0.31) [31]. In other studies, RA concentration data have not been collected regarding variables such as sex and age, although they were considered when logistic regression models were performed [38].

There was also no correlation between tobacco consumption and atRA concentration with respect to the group of patients with OL (*p* = 0.395), which again coincides with the results of Pérez-Sayáns et al. (*p* = 0.56) [31]. Regarding the treatment received from the moment of diagnosis, no statistically significant association was found with any of the three parameters (laser/follow-up/surgical excision) and the concentration of atRA (*p* = 0.063), although similarities were observed in the outcomes of patients who received laser therapy as opposed to those who were simply monitored for the control of their lesions. This fact is in line with what the literature indicates about the effectiveness of laser treatments, which describes quite high reappearance rates (up to 31%) [39], which would reflect the continuity of the precipitating factors to the appearance of lesions. ROC curve analysis revealed only modest discriminative ability of atRA concentrations to differentiate between the studied groups. The highest AUC was observed in the PVL group (AUC = 0.60), suggesting limited diagnostic potential. In contrast, the AUC values for the OL and control groups were below 0.50, indicating poor and even worse-than-chance classification performance (Figure 3). These findings highlight that, while statistically significant differences in RA concentrations were found between some groups—particularly between OL and PVL—atRA alone may not serve as a reliable standalone biomarker for diagnostic discrimination in this context.

Nowadays, it is well known that PVL becomes malignant in the form of certain types of carcinomas with higher survival rates, which have a warty appearance among the common clinical characteristics, are more recurrent, etc. [40]. The results of this study would support these premises, since the atRA concentrations of patients with PVL were very similar to healthy controls, which could perhaps be related to less aggressive behavior, and it could be thought that these lesions have affected molecular mechanisms and cells other than those that cause malignancy in OL. Thus, in the case of malignant transformation of an OL, survival is lower and is associated with risk factors different from those described for PVL [41].

This finding should be contextualized within the broader literature on RA levels in cancer and premalignant conditions. Previous studies have reported inconsistent results regarding RA concentration differences between cancer patients and healthy controls. While some investigations have demonstrated reduced circulating levels of RA in patients with established malignancies [30,31], others have found more nuanced relationships dependent on cancer type, stage, and patient characteristics. Several large-scale studies investigating retinoid metabolism in cancer have demonstrated more robust associations than we were able to detect. For example, Mongan and Gudas (2017) [42] conducted a comprehensive review of retinoid signaling in cancer, synthesizing data from multiple large cohorts that demonstrated consistent RA pathway alterations across various malignancies.

In the article by Pérez-Sayáns et al., patients with a greater tendency to have cancer are associated with a lower concentration of atRA compared to healthy patients [31], which is consistent with the results of the present study, although the mean atRA concentration of the patients who became malignant was 1.67 ± 0.49 pg/mL, compared to an average concentration of 4.25 ± 1.56 pg/mL in those that did not become malignant (*p* = 0.03) [31]. If we compare the atRA concentration values of both studies, it coincides that the lowest values would be those of patients with OSCC (1.67 ± 0.49 pg/mL), followed by patients with OL (2.17 ± 0.39 pg/mL) and lastly the healthy ones (4.7370 ± 1.5992 pg/mL in Pérez-Sayáns study, and 2.6580 ± 0.92032 pg/mL on this work). Although the discrepancy in concentration presented by the controls in one study compared to the other is striking, it must be considered that the sample size was smaller in the work of Pérez-Sayáns et al. and that the type of population was different, since the control group was matched with the relatives of the study patients and not with the specific OPMDs included here.

There are several studies, including randomized clinical trials and systematic reviews, in which a clinical improvement in OL lesions has been seen when intervening locally with retinoic acid or vitamin A [43,44]. The use of retinoic acid systemically over a period of 3 months also resulted in an improvement of up to 67% of the lesions by reducing their size [45]. However, other studies do not find significant differences between patients treated with chemopreventive agents and those who received a placebo [46]. In the case of PVL, the improvement is milder and less frequent, in only approximately one third of cases, with reappearances 2–3 months after treatment; these differences could be explained by the different evolutionary patterns that PVL presents with respect to OL. For all these reasons, there is still no evidence that there are truly effective treatments for OL and PVL to prevent their malignant transformation [19].

Despite the limitations in diagnostic utility as a standalone biomarker, these findings have several important translational implications. First, they provide a biological rationale for the observed clinical efficacy of topical retinoids in OL treatment, as reported in several clinical trials [43,44]. The specific RA deficiency in OL patients, contrasted with preserved levels in PVL, may explain the differential treatment responses observed clinically between these conditions.

Second, our results suggest that RA measurement could be valuable as part of a multi-biomarker panel rather than as an isolated diagnostic tool. Combined with other molecular markers of oral carcinogenesis, RA levels might contribute to risk stratification models that could inform treatment intensity and follow-up protocols.

Finally, the preservation of normal RA levels in PVL patients, despite their higher malignant potential, suggests distinct pathophysiological mechanisms between conventional OL and PVL. This finding supports the current clinical approach of treating these conditions as separate entities with different management protocols.

To strengthen the validity and generalizability of the findings, it is crucial to acknowledge several important limitations inherent to this pilot investigation. While our sample size (*n* = 36) was calculated a priori based on previous research and achieved adequate statistical power for the primary comparisons (>80% power for detecting observed effect sizes), the pilot nature of this study limits the scope of conclusions that can be drawn. As an exploratory investigation designed to establish proof-of-concept evidence for RA pathway alterations in oral premalignancy, this work provides a foundation for larger validation studies rather than definitive clinical guidance.

The exclusion of four samples due to out-of-range values (11% of the initial sample) may have introduced selection bias, particularly if these samples represented extreme phenotypes that could inform our understanding of the RA-oral lesion relationship. Future studies should employ more sensitive analytical methods to minimize sample loss and capture the full spectrum of RA concentrations.

The low number of malignant transformation cases (*n* = 3), all within the PVL group, significantly limits conclusions about disease progression and prognosis. This limitation is inherent to the cross-sectional design and the relatively short follow-up period typical of pilot studies. Larger longitudinal cohort studies with extended follow-up periods are essential to establish the predictive value of RA measurements for malignant transformation risk. Further studies comparing atRA levels across well-defined stages of PVL progression and OSCC subtypes are needed to substantiate this proposed carcinogenic sequence.

Several important confounding factors were not controlled for in this pilot study. Variations in lesion stage, duration, or treatment history, which could influence RA levels, were not systematically accounted for. Dietary intake and vitamin A metabolism, which can significantly influence plasma atRA concentrations, were not standardized or assessed, introducing potential inter-individual variability in RA levels. Additionally, genetic polymorphisms in RA metabolism genes (such as *CYP26A1* and *CYP26B1*) can alter atRA clearance and were not evaluated, potentially accounting for some of the observed variability.

The lower atRA levels observed in our healthy control group compared to other studies may reflect regional differences in study population, dietary patterns, or methodological variations. Environmental and dietary factors specific to Northern Spain, including regional dietary habits that may influence vitamin A intake, could contribute to these differences. Although sunlight exposure does not directly affect vitamin A levels, it may have indirect effects on retinoid metabolism through its role in skin physiology and related metabolic pathways.

The lack of strong associations between atRA levels and clinical parameters (sex, tobacco use, treatment modality) may stem from the limited sample size of this pilot study or insufficient sensitivity of our analytical approach. More sophisticated statistical models incorporating multiple potential confounders and larger datasets will be necessary to fully characterize these relationships.

## 5. Conclusions

This pilot study provides robust preliminary evidence that patients diagnosed with oral leukoplakia exhibit significantly reduced serum concentrations of all-trans retinoic acid (atRA), with an observed 18% decrease relative to healthy controls (2.17 ± 0.39 vs. 2.66 ± 0.92 pg/mL; *p* = 0.039), and a 22% decrease when compared to patients with proliferative verrucous leukoplakia (2.17 ± 0.39 vs. 2.64 ± 0.56 pg/mL; *p* = 0.009). These findings suggest a potential disruption of the retinoid signaling axis specifically associated with oral leukoplakia, which may reflect early molecular alterations predisposing to malignant transformation.

While the ROC curve analysis demonstrated limited diagnostic performance (AUC 0.45–0.60), insufficient for clinical application as a standalone biomarker, the consistent and biologically plausible trend toward atRA depletion in oral leukoplakia supports the hypothesis that atRA pathway dysregulation may be involved in the etiopathogenesis of this condition. Importantly, the oral leukoplakia group in this study represents a clinically heterogeneous but pathologically significant subset of oral potentially malignant disorders, emphasizing the need for improved molecular characterization.

These data offer important proof-of-concept support for the involvement of retinoic acid metabolism in early oral carcinogenesis, particularly within the oral leukoplakia phenotype. Future studies with larger sample sizes, longitudinal designs, and integrative molecular profiling are essential to validate these observations, delineate the mechanistic underpinnings of atRA depletion, and explore its prognostic and therapeutic relevance. Ultimately, clarifying the role of atRA in homogenous and proliferative leukoplakias may contribute to the development of more precise risk stratification tools and targeted chemopreventive strategies in the management of OPMDs.

## Figures and Tables

**Table 1:** Clinical and Demographic Characteristics of the Study Population (N = 36).

Clinical Variable	N (%)
**Group**	
Proliferative Verrucous Leukoplakia (PVL)	9 (25.0%)
Oral Leukoplakia (OL)	10 (27.8%)
Control	17 (47.2%)
**Sex**	
Male	15 (41.7%)
Female	21 (58.3%)
**Tobacco Use**	
Non-smoker	21 (58.3%)
Smoker	5 (13.9%)
Ex-smoker	10 (27.8%)
**Alcohol Consumption**	
Non-drinkers	18 (50.0%)
Moderate drinkers	12 (33.3%)
Heavy drinkers	6 (16.7%)
**Malignant Transformation**	
No	17 (85.0%)
Yes	3 (15.0%)

**Table 2:** Detailed Patient Characteristics, Clinical Data, and Histopathological diagnosis (N = 36).

Patient ID	Gender	Smoking Status	Alcohol Use	Anatomical Site	Clinical Diagnosis	Histopathological Diagnosis
**PVL Group (N = 9)**						
PVL001	Female	Smoker	Drinker	Buccal Mucosa, Lateral tongue	PVL	EHK
PVL002	Female	Smoker	Drinker	Buccal Mucosa, Lateral tongue	PVL	Moderate OED
PVL003	Male	Non-smoker	Drinker	Alveolar ridge, Lateral tongue	PVL	Mild OED
PVL004	Female	Non-smoker	Non-drinker	Lateral tongue, Gingiva	PVL	Mild OED
**PVL005** *	Female	Non-smoker	Non-drinker	Gingiva, Soft palate	PVL	Severe OED
PVL006	Female	Non-smoker	Non-drinker	Gingiva, Buccal mucosa	PVL	EHK
**PVL007** *	Female	Smoker	Non-drinker	Alveolar ridge	PVL	Severe OED
PVL008	Male	Smoker	Drinker	Gingiva, Soft palate	PVL	Mild OED
**PVL009** *	Female	Smoker	Non-drinker	Buccal mucosa, Gingiva	PVL	Severe OED
**OL Group (N = 10)**						
OL001	Female	Non-smoker	Non-drinker	Lateral tongue	OL	Moderate OED
OL002	Female	Smoker	Non-drinker	Lateral tongue	OL	Mild OED
OL003	Female	Non-smoker	Non-drinker	Ventral tongue	OL	Moderate OED
OL004	Male	Smoker	Non-drinker	Buccal mucosa	OL	Mild OED
OL005	Female	Smoker	Non-drinker	Buccal mucosa	OL	Mild OED
OL006	Female	Non-smoker	Non-drinker	Buccal mucosa	OL	Moderate OED
OL007	Female	Smoker	Non-drinker	Lateral tongue	OL	Mild OED
OL008	Male	Smoker	Non-drinker	Buccal mucosa	OL	Mild OED
OL009	Male	Smoker	Drinker	Floor of mouth	OL	Mild OED
OL010	Male	Smoker	Non-drinker	Lateral tongue	OL	Moderate OED
**Control Group (N = 17)**						
CTRL001	Female	Non-smoker	Non-drinker	–	Control	Normal
CTRL002	Male	Ex-smoker	Moderate drinker	–	Control	Normal
CTRL003	Female	Non-smoker	Non-drinker	–	Control	Normal
CTRL004	Male	Ex-smoker	Non-drinker	–	Control	Normal
CTRL005	Female	Non-smoker	Moderate drinker	–	Control	Normal
CTRL006	Male	Smoker	Drinker	–	Control	Normal
CTRL007	Female	Non-smoker	Non-drinker	–	Control	Normal
CTRL008	Male	Ex-smoker	Moderate drinker	–	Control	Normal
CTRL009	Female	Non-smoker	Non-drinker	–	Control	Normal
CTRL010	Male	Ex-smoker	Non-drinker	–	Control	Normal
CTRL011	Female	Smoker	Moderate drinker	–	Control	Normal
CTRL012	Male	Ex-smoker	Drinker	–	Control	Normal
CTRL013	Female	Non-smoker	Non-drinker	–	Control	Normal
CTRL014	Male	Ex-smoker	Moderate drinker	–	Control	Normal
CTRL015	Female	Non-smoker	Non-drinker	–	Control	Normal
CTRL016	Male	Smoker	Drinker	–	Control	Normal
CTRL017	Female	Smoker	Non-drinker	–	Control	Normal

Abbreviations: PVL: Proliferative Verrucous Leukoplakia; OL: Oral Leukoplakia; OED: Oral Epithelial Dysplasia; EHK: Epithelial Hyperkeratosis; *: Patients who developed malignant transformation.

**Table 3:** atRA concentration in patients according to clinical variables. The values marked in bold correspond to those that show a statistically significant association (*p* < 0.05).

Variable	OL	PVL	Control	*p* Value
**Group comparison**				
Overall (N)	18	14	30	
Mean (SD)	2.17 (0.39)	2.64 (0.55)	2.65 (0.92)	**0.009** *
Range	1.75–3.09	1.95–3.95	1.45–4.76	**0.039** ^†^, 0.953 ^‡^
**Sex**				
Male	1.69 (0.67) [N = 5]	2.23 (0.51) [N = 3]	2.82 (0.88) [N = 7]	0.105
Female	2.31 (0.32) [N = 5]	2.41 (0.84) [N = 6]	2.24 (0.98) [N = 10]	0.743, 0.231
**Tobacco**				
Non-smoker	2.05 (0.21) [N = 5]	2.29 (0.99) [N = 5]	2.54 (1.14) [N = 11]	0.329
Smoker	1.44 (1.34) [N = 2]	2.33 (0.09) [N = 3]	-	0.892
Ex-smoker	2.28 (0.41) [N = 3]	2.72 [N = 1]	2.38 (0.57) [N = 6]	0.756
**Alcohol**				
Non-drinker	2.11 (0.39) [N = 6]	2.52 (0.23) [N = 4]	2.48 (1.10) [N = 9]	0.478
Moderate drinker	1.95 (0.18) [N = 2]	2.81 (0.41) [N = 3]	2.77 (0.74) [N = 6]	0.387
Heavy drinker	2.40 (0.97) [N = 2]	2.71 (0.55) [N = 2]	2.90 (0.56) [N = 2]	0.421
**Treatment**				
Follow-up	2.03 (0.72) [N = 7]	1.99 (0.42) [N = 2]	-	0.813
Laser	1.92 (0.13) [N = 3]	2.24 (1.05) [N = 3]	-	0.629
Exeresis	-	2.62 (0.61) [N = 4]	-	

* OL vs. PVL; ^†^ OL vs. Control; ^‡^ PVL vs. Control. Bold values indicate statistical significance (*p* < 0.05).

## Data Availability

The data presented in this study are available on request from the corresponding author.
